# Use of Molecular Epidemiology to Inform Response to a Hepatitis A Outbreak — Los Angeles County, California, October 2018–April 2019

**DOI:** 10.15585/mmwr.mm6926a3

**Published:** 2020-07-03

**Authors:** Meredith Haddix, Rachel Civen, Jill K. Hacker, Will Probert, Sarah New, Nicole Green, Peera Hemarajata, Prabhu Gounder

**Affiliations:** ^1^Acute Communicable Disease Control Program, Los Angeles County Department of Public Health, California; ^2^Community Health Services Program, Los Angeles County Department of Public Health, California; ^3^Viral and Rickettsial Disease Laboratory, California Department of Public Health; ^4^Immunization Branch, California Department of Public Health; ^5^Public Health Laboratory, Los Angeles County Department of Public Health, California.

Los Angeles County comprises 4,058 square miles and is home to approximately 10 million residents (*1*), an estimated 59,000 (0.6%) of whom experience homelessness on a given night (*2*). In late 2018, Los Angeles County Department of Public Health (LAC DPH) was notified of a case of hepatitis A virus (HAV) infection in a person experiencing homelessness. LAC DPH conducted an investigation to determine the source of infection, identify additional cases, and identify contacts for postexposure prophylaxis (PEP). Over the next week, LAC DPH identified two additional hepatitis A cases in persons experiencing homelessness who knew one another socially and were known to congregate at a specific street intersection. To identify and respond rapidly to additional outbreak-associated cases, LAC DPH implemented enhanced surveillance procedures, including immediately obtaining specimens for molecular testing from all patients with suspected hepatitis A in the same geographic area. Enhanced surveillance identified four additional cases in persons linked to a senior living campus within two blocks of the intersection where the initial three patients reported congregating. These four cases were linked to the cluster in persons experiencing homelessness through HAV genotyping. Overall, DPH identified seven outbreak-associated hepatitis A cases during October 2018–January 2019. The DPH response to this community hepatitis A outbreak included conducting vaccination outreach to persons at risk, conducting environmental health outreach to restaurants in the outbreak area, and issuing health care provider alerts about the increased occurrence of hepatitis A. Implementation of near real-time molecular testing can improve hepatitis A outbreak responses by confirming HAV infections, linking additional cases to the outbreak, and informing the targeting of prevention efforts.

## Investigation and Results

Health care providers and clinical laboratories are mandated to report hepatitis A cases within one working day of identification.* DPH staff members investigate reported hepatitis A cases to determine whether they meet the national surveillance acute hepatitis A case definition. In 2018, a confirmed case of acute HAV infection was defined as illness occurring in a person with 1) a discrete onset of hepatitis symptoms, 2) jaundice or elevated alanine aminotransferase (ALT) or aspartate aminotransferase (AST), and 3) reactive anti-HAV immunoglobulin (Ig) M antibody (*3*). Patients with confirmed HAV infection are interviewed using a standard questionnaire to assess risk factors and to identify contacts who can be offered PEP.

On November 10, 2018, an acute hepatitis A case was reported to DPH in a person experiencing homelessness who used methamphetamines (patient A) (Table) (Figure). Medical records review indicated that patient A was transported to the emergency department of hospital A by ambulance from intersection X but left the hospital against medical advice and could not be located by DPH for interview. Patient A did not report nausea, vomiting, or abdominal pain but did have left flank pain, fever, an elevated ALT and a positive anti-HAV IgM test result. Another person experiencing homelessness who reported methamphetamine use (patient B) was evaluated 3 days later at hospital B with a 3-day history of nausea and abdominal pain. The patient received a diagnosis of HAV infection, and the diagnosis was reported to DPH on November 14, 2018.

**TABLE:** Demographic and clinical characteristics of patients with suspected outbreak-associated hepatitis A virus (HAV) infection — Los Angeles County, California, October 2018–April 2019*

Characteristic	Patient											
	A	B	C	D^†^	E	F^†^	G	H	I^†^	J	K^†^	L^†^
Report date^§^	Nov 11, 2018	Nov 14, 2018	Oct 18, 2018	Nov 20, 2018	Dec 5, 2018	Dec 9, 2018	Dec 11, 2018	Dec 21, 2018	Jan 7, 2019	Jan 13, 2019	Feb 5, 2019	Mar 6, 2019
Age group (yrs)	35–44	35–44	35–44	18–34	55–64	35–44	≥75	18–34	65–74	≥75	18–34	45–54
Jaundice	No	No	No	Yes	Yes	No	No	Yes	Yes	Yes	Yes	Yes
Symptoms^¶^	Yes	Yes	Yes	Yes	Yes	Yes	No	Yes	Yes	No	Yes	Yes
Hospitalized	No	Yes	Yes	Yes	Yes	Yes	No	Yes	Yes	Yes	No	No
HAV IgM+	Yes	Yes	Yes	Yes	Yes	Yes	Yes	Yes	Yes	Yes	Yes	Yes
ALT >200	Yes	Yes	Yes	Yes	Yes	Yes	No	Yes	Yes	Yes	Yes	Yes
TBil ≥3.0	No	No	No	Yes	Yes	No	No	Yes	Yes	Yes	Yes	Yes
Genotype	IB	IB	Unknown	Unknown	IB	Unknown	Unknown	IB	IA	IB	IB	Unknown
Strain	CA Cls A	CA Cls A	Unknown	Unknown	CA Cls A	Unknown	Unknown	CA Cls A	Unique	CA Cls A	A16MI Cls 2	Unknown
Homeless	Yes	Yes	Yes	No	No	No	No	No	No	No	Yes	No
Illegal drug use**	Yes	Yes	Yes	Yes	No	No	No	No	No	No	Yes	No
Linked to senior living campus	No	No	No	No	Yes (visitor)	No	Yes (resident)	Yes (staff member)	No	Yes (resident)	No	No
Epi-link to outbreak case	Yes	Yes	Yes	No	Yes	No	Yes	Yes	No	Yes	No	No
Met surveillance case definition^††^	Yes	Yes	Yes	Yes	Yes	Yes	No	Yes	Yes	No	Yes	Yes
Met outbreak case definition^§§^	Yes	Yes	Yes	No	Yes	No	Yes	Yes	No	Yes	No	No

**Abbreviations:** ALT = alanine amino transferase; CA = California; Cls = cluster; Epi-link = epidemiologic link; HAV IgM+ = positive immunoglobulin M antibody against HAV; TBil = total bilirubin.

* Los Angeles County Department of Public Health declared the outbreak over after 100 days without additional outbreak-associated hepatitis A cases (representing two HAV infection incubation periods)

^†^ Not outbreak-associated.

^§^ Dates have been shifted to preserve patient confidentiality.

^¶^ Symptoms compatible with acute HAV infection, including fever, headache, malaise, anorexia, nausea, vomiting, diarrhea, and abdominal pain.

** Includes illegal drug use in the state of California, including use of methamphetamines, cocaine, heroin, and prescription opioids that have not been prescribed to the user. Does not include marijuana use.

^††^ National surveillance acute hepatitis A case definition in 2018: acute illness with discrete onset of symptoms consistent with acute viral hepatitis, jaundice or elevated ALT or aspartate aminotransferase, and IgM antibody to hepatitis A virus (anti-HAV) positive.

^§§^ Hepatitis A infections in persons residing or spending time in outbreak area and infection caused by HAV genotype IB, CA Cls A, or if no genotype available, epidemiologic link to outbreak case.

**FIGURE:**
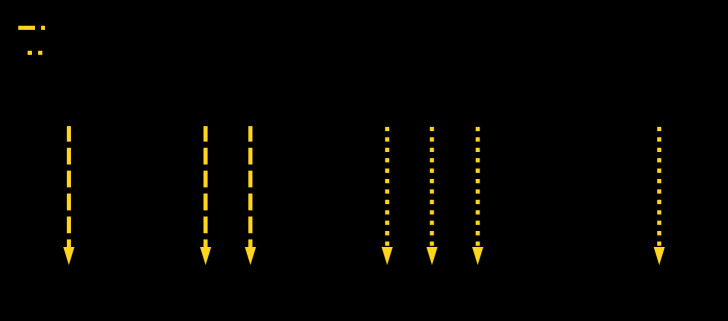
Timeline of confirmed outbreak-associated* hepatitis A virus (HAV) cases and public health response — Los Angeles County, California, October 2018–January 2019^†,§^ * Outbreak cases were defined as HAV infections occurring in persons who 1) resided or spent time in the outbreak area and 2) either had infections caused by HAV genotype IB CA cluster A strain or were epidemiologically linked to a person infected with the outbreak strain. ^†^ Dates have been shifted to preserve patient confidentiality. ^§^ Enhanced surveillance continued until the outbreak was declared over in April 2019. Los Angeles County Department of Public Health declared the outbreak over after 100 days without additional outbreak-associated HAV cases (representing two HAV infection incubation periods).

Upon DPH interview, patient B reported using public restrooms located in restaurants and stores at intersection X and named patient A as a contact who was ill. Patient B also named an acquaintance (patient C) with acute hepatitis A who had been reported to DPH 1 month earlier and could not be interviewed when originally reported. Patient B stated that patient C also frequented intersection X, lived unsheltered nearby, and had shared drug equipment with patient A. Serum from patients A and B were sent to the California Department of Public Health (CDPH) Viral and Rickettsial Disease Laboratory for sequence-based genotyping targeting a segment of the VP1-P2B genomic region (*4*). A genotype IB sequence (CA Cluster [Cls] A) matching a recent outbreak strain, USA/2017/V17S07250 (GenBank accession number MH577310), was detected in both specimens.

After identifying hepatitis A cases in three epidemiologically linked persons, DPH implemented enhanced surveillance procedures to rapidly detect and respond to any secondary cases. Enhanced surveillance was conducted within a 50-square mile area bounded by four major freeways, on the assumption that movement of persons might be constrained by these roadways. DPH immediately attempted to obtain and hold all anti-HAV IgM-positive serum specimens from patients residing within the outbreak area; serum specimens from persons who met the national surveillance acute hepatitis A case definition or were epidemiologically linked to a confirmed case were sent to CDPH for molecular testing. These procedures were maintained until 100 days had elapsed without additional outbreak-associated hepatitis A cases (representing twice the HAV infection incubation period).

Outbreak-associated cases were defined as HAV infections occurring in persons who 1) resided or spent time in the outbreak area during October 15, 2018–April 29, 2019 and 2) either had infections caused by the HAV genotype IB CA Cls A strain or were epidemiologically linked to a person infected with the outbreak strain. DPH staff members interviewed persons linked to the outbreak with a supplementary outbreak-specific questionnaire to 1) assess any additional sources of HAV exposure, 2) identify potentially ill persons who might not have sought medical care, and 3) identify areas where ill persons congregated during the infectious period to guide prevention outreach efforts.

Among the 19 anti-HAV IgM-positive cases reported to DPH during November 10, 2018–April 29, 2019, from the outbreak area, 10 did not meet the national surveillance acute hepatitis A case definition (surveillance case definition) or outbreak hepatitis A case definition (outbreak case definition). Five patients (D, F, I, K, and L) did meet the surveillance case definition but did not meet the outbreak case definition (Table), two (E and H) met both the surveillance and outbreak case definitions, and two (G and J) met the outbreak case definition only. Patient K’s illness was initially classified as an outbreak-associated case because the patient reported both homelessness and methamphetamine use and resided near intersection X during the incubation period. However, genotyping subsequently revealed that patient K was infected with a different HAV strain, so the case was reclassified as not outbreak-associated.

The four outbreak-associated cases (in patients E, G, H, and J) identified after the initial three (in patients A, B, and C) occurred in persons who did not report homelessness or illegal drug use (Table) (Figure). These four cases were linked to a senior living campus as either residents (two), a staff member (one), or a visitor (one). Serum for molecular testing was available for patients E, H, and J; all were HAV genotype IB, CA Cls A. Patients G and J did not meet the surveillance case definition because they did not have symptoms compatible with acute hepatitis. Patient J, however, had an infection caused by the outbreak strain and patient G was epidemiologically linked to patient E, who was infected with the outbreak strain. All four patients were interviewed to assess potential common exposures to patients A, B, and C. Patients G and H reported patronizing businesses in intersection X.

DPH maintained enhanced surveillance for 100 days following the last day of patient J’s infectious period and identified no additional outbreak cases. Five of the seven persons with outbreak-associated HAV infection were hospitalized (Table); none died. DPH declared the outbreak over on April 29, 2019.

## Public Health Response

After identification of cases of HAV infection in persons experiencing homelessness, DPH sent a health alert to Los Angeles County health care personnel advising them to remain vigilant for hepatitis A in persons experiencing homelessness or using drugs and to immediately notify DPH of any suspected hepatitis A cases.

Based on responses of patients with outbreak-associated cases to the outbreak-specific questionnaire, DPH targeted hepatitis A vaccination efforts to reach persons with similar risk factors in the geographic area where patients A, B, and C had dwelt beginning November 22 (week 47) (*5*). After identification of a confirmed outbreak-associated case in a visitor to the senior living campus (patient E) and a suspected case in the resident visited by patient E (and before identification of the other two outbreak-associated cases), hepatitis A vaccination clinics were held for residents and staff members beginning the week of December 17 (week 51) (Figure). In total, 857 hepatitis A vaccine doses were provided at the senior living campus, drug treatment centers, food pantries, and homeless shelters during November 22, 2018–March 13, 2019.

Environmental health staff members visited 22 restaurants near intersection X and the senior living campus to assess sanitation and hygiene procedures and provide education. They also sent an email with information about hepatitis A and sanitation to all restaurants within the two ZIP codes where patients A, B, and C spent time during their infectious periods.

## Discussion

A hepatitis A outbreak occurred in Los Angeles County among persons with a history of homelessness and illegal drug use and among persons residing in the same geographic area who had no identifiable hepatitis A risk factors (*6*,*7*). Since 2016, multiple large and ongoing hepatitis A outbreaks have occurred in the United States, disproportionately affecting persons with a history of homelessness or drug use (*7*,*8*). Genotyping has been used to retrospectively characterize the HAV strains causing the outbreaks (*8*). This report describes the use of rapid molecular testing in LAC to guide an ongoing community hepatitis A outbreak response by confirming infection, linking cases to the outbreak, and informing prevention outreach efforts.

Genotyping improved outbreak characterization and response in several ways. First, genotyping helped to narrow the scope of LAC DPH response activities by excluding cases identified as having a nonmatching strain. For example, patient K would have been considered part of the outbreak based on epidemiologic factors alone. Because patient K’s HAV strain did not match the outbreak strain, DPH was able to reduce the period of enhanced surveillance by approximately 3 weeks and redirect efforts toward investigating an independent chain of transmission. Second, the identification of matching strains helped to link cases that did not have any other apparent epidemiologic connections, leading DPH to hypothesize that transmission occurred through exposure to common community spaces (such as public restrooms) and conduct outreach to local businesses to encourage enhanced environmental sanitation procedures. Finally, molecular testing confirmed infections in persons who did not meet the national surveillance acute hepatitis A case definition. Confirming these additional cases provided LAC DPH with an opportunity to implement timely control measures and potentially prevented additional cases. 

Obtaining specimens for HAV genotyping is challenging. Serum intended for sequencing must be appropriately processed and frozen within 72 hours of collection, which commercial laboratories typically only do upon request. The routine hepatitis A surveillance case reporting and investigation process can take >72 hours, so often serum is no longer available by the time a case is confirmed. Therefore, as part of the enhanced surveillance efforts, DPH immediately contacted laboratories to obtain any anti-HAV IgM-positive serum within the outbreak area while investigating to determine if the specimen met criteria for molecular testing. The increased resource requirement for the expanded effort (in terms of staff member time and shipping costs) was manageable because it was limited to a defined period and within a specific geographic area. However, in the setting of widespread community transmission, such an approach might not be feasible.

The findings in the report are subject to at least three limitations. First, the CA Cls A strain is a commonly identified cause of many national hepatitis A outbreaks, indicating that it might be an endemic strain (*4*). Therefore, it is possible that the outbreak-associated cases linked to the senior living campus represent a chain of transmission distinct from the cases among persons experiencing homelessness or using drugs. Second, HAV strain results must be interpreted in the context of the epidemiologic information. The interpretation of genotyping results from this investigation might have been limited by patients’ not disclosing certain risk factors or exposures. Finally, the sensitivity of molecular testing for confirming hepatitis A can be reduced by improper specimen handling or if specimens are obtained after a substantial time has elapsed since symptom onset. Thus, it is possible that some anti-HAV IgM-positive cases were misclassified as false-positive case reports.

This outbreak response illustrates the value of using rapid HAV molecular testing to characterize an outbreak and guide the public health response to contain the outbreak. HAV genotyping can be helpful in identifying and interrupting the chain of transmission early in an outbreak when there are few cases. HAV genotyping in other contexts might provide additional insights into its optimal use for outbreak prevention and control.

**Summary**

**What is already known about this topic?**

Sequence-based genotyping has been valuable for retrospectively characterizing and identifying the potential sources of hepatitis A outbreaks.

**What is added by this report?**

After identification of a case of hepatitis A in a person experiencing homelessness, Los Angeles County implemented enhanced surveillance and near real-time molecular testing, which identified two additional cases in homeless persons and four cases in a senior living campus; genotyping results linked the two clusters and informed the outbreak response.

**What are the implications for public health practice?**

Conducting sequence-based genotyping of hepatitis A virus strains, especially early in an outbreak when there are few cases, can result in targeted and timelier implementation of effective prevention and control efforts.

## Footnotes

* Per Title 17 of the California Code of Regulations. Cal. Code Regs. tit. 17, sect. 2500, sect. 2505 (2020).
